# Eosinophilic Angiocentric Fibrosis of the Nasal Septum

**DOI:** 10.1155/2013/267285

**Published:** 2013-03-24

**Authors:** Yunchuan Li, Honggang Liu, Demin Han, Hongrui Zang, Tong Wang, Bin Hu

**Affiliations:** ^1^Department of Otolaryngology, The Key Discipline of Ministry of Education, Beijing Tongren Hospital, Capital Medical University, Beijing 100730, China; ^2^Department of Pathology, Beijing Tongren Hospital, Capital Medical University, Beijing 100730, China; ^3^Institute of Otolaryngology, Beijing, China

## Abstract

*Background*. Eosinophilic angiocentric fibrosis (EAF) is a rare benign condition of unknown aetiology that causes stenosis of the upper respiratory tract. It is most commonly found at the nasal septum and sinus mucosa causing mucosal thickening and nasal obstructive symptoms. The diagnosis is mainly based on characteristic histologic findings. *Case Report*. A 27-year-old young woman presented with a slow growing mass at her anterior nasal septum for over eight years. She complained of persistent nasal obstruction, epistaxis, sometimes diffused facial pain, and chronic headache. 3 years ago, the tumor was partially resected for ventilation and a nasal septum perforation was left. Imaging findings indicated soft-tissue thickening of the anterior part of septum and adjacent lateral nasal walls. Pathological examination showed numerous inflammatory cells infiltrates containing eosinophils, fibroinflammatory lesion with a whorled appearance fibrosis which typically surrounded vessels. A diagnosis of eosinophilic angiocentric fibrosis was made. All laboratory tests were unremarkable. Skin prick test was positive. The tumor-like lesion was totally resected. *Conclusions*. EAF is a rare benign and progressive disorder causing destruction. Combined with radiological imaging of EAF historical findings contribute to the diagnosis. It is important to prevent tumor from recurrence by total resection of the lesion.

## 1. Introduction

Eosinophilic angiocentric fibrosis (EAF) is a rare, benign condition of unknown aetiology which may cause local tissue progressive destruction [1]. It mainly involves the sinonasal tract and is especially common at the nasal septum. EAF typically presents in young to middle-aged females. Most of the patients complain of progressive sinonasal obstructive with a tumor-like lesion. The etiology of EAF is unknown, and the diagnosis is mainly based on histologic findings. The histologic features include perivascular inflammatory cell infiltration (mainly eosinophils). The eosinophils infiltration is gradually replaced by the progressive fibrosis lesion with “onion-skin” pattern around small blood vessels [1–6]. 

It was first described by Holmes and Panje in 1983 who reported a case of so-called ‘‘intranasal granuloma faciale” [7]. After two years, Roberts and McCann reported two female patients with an unusual stenosing lesion involving the upper respiratory. They gave a descriptive diagnosis according to the histologic findings: eosinophilic angiocentric fibrosis [8]. Until now, 51 patients diagnosed with EAF have been reported in the English literature including our case [1–33]. The main occurrences of EAF originated from the nasal cavity (46/51, 90.2%) and the most common symptom of EAF was progressive nasal obstruction [1–6, 13–15, 17, 19, 22, 23, 26, 28, 30, 32]. The nasal septum (32/51, 62.7%) was the most common involvement site, including 12 patients' tumor like lesion extension into the lateral nasal wall and nasal base (12/32, 37.5%) [9, 10, 30]. The lesion also might arise from the lateral nasal wall and it might demonstrate an irregular shape and ill-defined margins (14/51, 27.5%) [5, 17, 32]. Five cases (5/51, 9.8%) who complained of diplopia and epiphora showed orbit, lacrimal gland and other adjacent anatomic site involvement based on CT and MR imaging findings [13, 17, 18]. Although rare, there still was report on the involvement of the larynx and trachea by EAF [34]. 

We described a case of a young woman with primary nasal septum tumor-like lesion which was diagnosed as EAF based on historical findings and gave a literature review of EAF.

## 2. Case Report

### 2.1. Patient Information

A 27-year-old otherwise healthy young woman presented with a slow growing mass at her anterior nasal cavity for over eight years. Her symptoms included persistent nasal obstruction, recurrent nasal discharge and epistaxis, sometimes diffused facial pain, and chronic headache. The lesion completely obstructed both nostrils three years ago. She sought help for nasal ventilation in another ENT center. Submucous thickening tissue was locally resected and the involved anterior nasal septum cartilage was also partially removed. Histopathological examination of the biopsy indicated fibrous tissue with hyaline degeneration. Two years ago, bilateral alternative nasal obstruction reoccurred and got worse and worse. Physical examination showed that the patient had a broad nasal bridge. A large submucosal thickening of the anterior septum felt solid masses on palpation. There were no cutaneous lesions of nasal vestibule or her face. Endoscopic examination showed an anterior nasal septum perforation which might be due to the last surgery.

### 2.2. Radiological Examination

Imaging findings showed soft-tissue thickening of the anterior part of septum and adjacent lateral nasal walls. On nonenhanced CT, the lesions appeared homogeneously isoattenuated to gray matter. Perforation of the anterior nasal septum was identified with its size approximately 1.3 × 1 cm. Bone window showed localized and discontinuous oppressive thinning of the lower edge of frontal process of maxilla (P1). The tumor lesion involved soft tissues of piriform aperture and is close to the anterior wall ethmoid bone. From the MRI images, the tumor lesion was located at the anterior 1/3 of the septum and the adjacent lateral nasal walls, bilateral anterior inferior turbinate. The inner side of upper lateral nasal cartilage was isointense on the T1-weighted image while being hypointense on the T2-weighted image (P2). Lacrimal sac and nasolacrimal duct were normal. Chest X-ray found no nodule or other abnormality.

### 2.3. Pathological Findings

Pathological examination showed that numerous inflammatory cells were infiltrated with eosinophils, plasma cells, and lymphocytes. The predominant cells were eosinophils. Nasal biopsy revealed fibro-inflammatory lesions with a whorled appearance fibrosis which typically surrounded vessels. It also showed a concentric “onion-skin” fibrosis formed without any small-caliber vessels in its center. There were still some eosinophils infiltrations. Fibrinoid necrosis or true granuloma formation was absent in these biopsy tissues.

### 2.4. Laboratory Investigations

Whole blood neutrophil count was slightly lower. Other blood routine examinations (Hct, Hb, and plt), blood biochemistry, erythrocyte sedimentation rate (ESR), and coagulation parameter were in normal ranges. Serum immunological examination indicators were in normal ranges except that IgA slightly increased to 613 mg/dL (82–453 mg/dL). Antinuclear antibody (ANA) and anti-double-stranded DNA (anti-ds-DNA) antibody were negative. Both anti-PR3 antibody (c-ANCA type) and anti-MPO antibody (p-ANCA type) were lower than 20 RU/ml. There was an excess of skin prick test with positivity to dandelion (++), dermatophagoides farine (++), and dermatophagoides pteronyssinus (++). Serum total IgE increased its level to 315 KU/L (reference value <60 KU/L). 

### 2.5. Treatment and Followup

Under general anesthesia, endoscopic surgery was performed with a 3 cm-long arc-shaped incision on the skin of the nasal vestibule from the lateral nasal cartilage to the septal cartilage. The masses profile was white and hypovascular. Perichondrium was stripped using periosteal elevator and the masses were totally fished out until the surrounding normal mucosa could be clearly identified. Endoscopic observation indicated the greater alar cartilage and septodorsal cartilage were partially involved and destroyed. Then the cartilage lesions were totally resected and the white formless tumor was fished out. The opposite nasal cavity was performed with the same procedures. The anterior nasal septum perforation, left without reconstruction. Two silicious tubes for dilation were placed from the anterior nares through the common meatus for three months. A light nasal pack was placed to safeguard against large clot accumulation. Antibiotics and glucocorticoid were used intravenously to prevent infection and alleviate edema. We recommended long-term intranasal topical corticosteroids and followup every three months. Two years after surgery, there was no sign of local recurrence and distant metastasis. 

## 3. Discussion

EAF is a rare benign disorder which is classified as a chronic fibrosing vasculitis. The underlying aetiology and pathophysiology are unclear. It mainly affects the sinonasal tract of young to middle-age patients leading to upper airways obstruction. To date, fifty-one cases have been described and the nasal septum was the most common affected location. There was not an apparent sex predilection judging from the review of all the current English literature (male : female = 20 : 31) [1–34].

**Figure 1:**
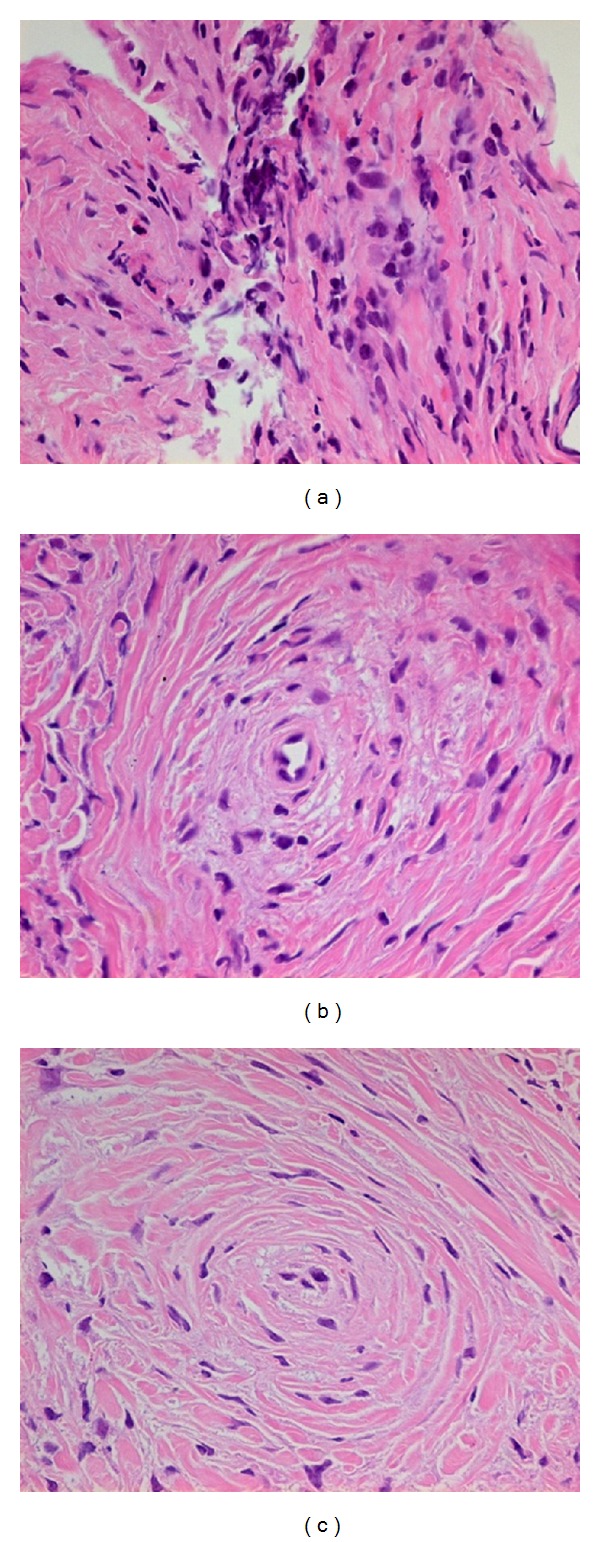
(a) The early inflammatory lesion. It shows the dense inflammatory infiltrate with numerous eosinophils on a background of chronic inflammatory cells. (b) A fibroinflammatory lesion. It is characterized by a whorled appearance perivascular fibrosis which typically surrounded vessels at the center. The predominant infiltration cells are eosinophils. (c) A nodule with onion-skin-type perivascular fibrosis. There might be capillaries and venules in the center of whorly thickening fibrous. Now the blood vessel lumen is obliterated. The inflammatory infiltrate is scanty, but eosinophils remain in the lesion.

The EAF is a historical diagnosis with two stages which has no clear boundary. It is characterized by progressing from an early eosinophil-rich perivascular fibrosing inflammatory lesion to a late dense perivascular “onion-skin” fibrosis formation with decreased inflammatory infiltration [1, 2, 4–6, 11, 12, 14, 18, 22, 26, 27, 29, 30, 32]. It is likely to see both early and later stages simultaneously at a single biopsy (Figure 1(b)). Dense eosinophilic infiltration accompanied by plasma cells and lymphocytes around the capillaries and venules generally appears in the early lesion (Figure 1(a)). In contrast, the late lesion shows numerous whorly thickening fibrous with a narrow or occlusive capillaries and/or venules at its center. The inflammatory infiltrate is scanty, but eosinophilsmightremain in the later stage (Figure 1(c)). 

**Figure 2:**
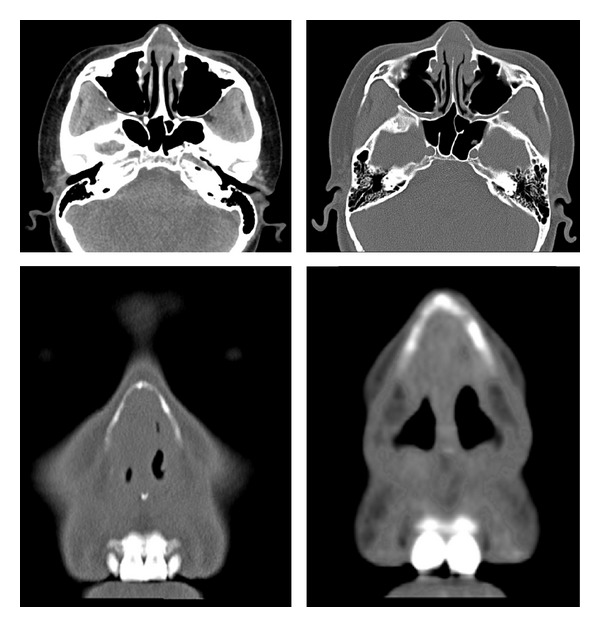
They indicate localized and discontinuous oppressive thinning of the lower edge of frontal process of maxilla.

**Figure 3:**
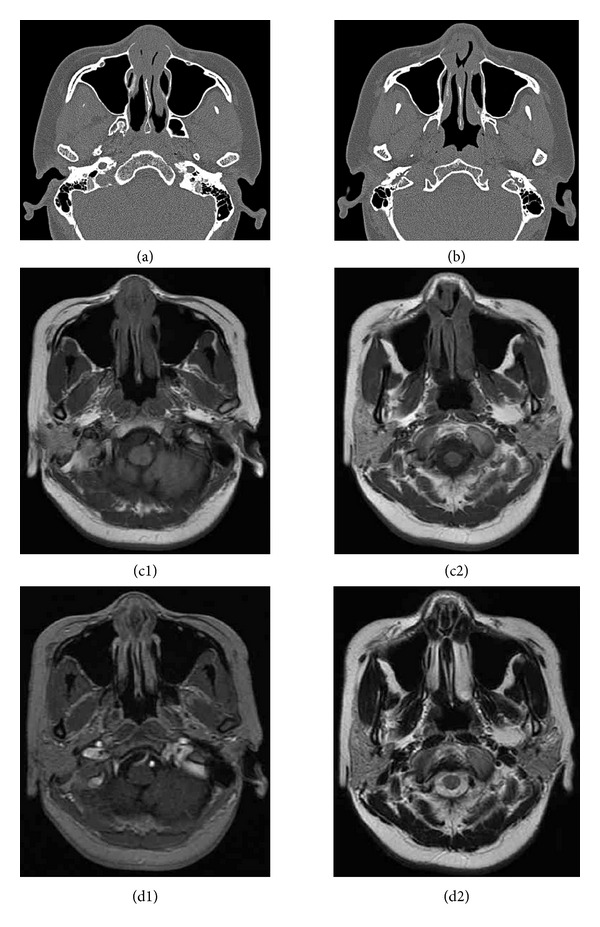
(a) The lesion appears to have homogeneous isodensity to gray matter on the nonenhanced CT. (b) The anterior nasal septum perforation was identified with its size approximately 1.3∗1 cm. (c1, c2) On the T1-weighted image, the anterior part of the septum and adjacent lateral nasal wall appear to be isointense to gray matter. (d1, d2) On the T2-weighted image, the anterior septum and bilateral headend of inferior turbinate are hypointense.

On non-enhanced CT, the lesion appeared to have homogeneous isodensity to gray matter and it may cause adjacent bone remodeling, thinning, and slight absorption with the lesion progress [10]. In our case, there was slight bony thinning in the lower edge of the frontal process of the maxilla (Figure 2). EAF was generally isointense relative to gray matter on T1-weighted images, with moderate inhomogeneous enhancement after contrast administration. The late stage with whorled “onion-skin” collagenous tissue seemed to be related to low signal-intensity foci on the T2-weighted image (Figure 3) [10]. 

Currently, the exact etiology of EAF remains unclear. Allergy, atopy, and trauma have been considered as predisposing factors [13, 16, 18, 27]. Our young female patient received a previous surgery only for ventilation due to preoperative indefinite diagnosis. It might be the incomplete excision that speeds up the tumor growth. There was no consensus whether allergy caused EAF or not. Allergic rhinitis is initiated by nasal mucosal infiltration with mast cells, basophils, and eosinophils. Our patient was allergic to dust mites and had higher serum total IgE levels eosinophils. It indicated that her allergic background might underlie allergic rhinitis and EAF. Although, allergic rhinitis is relatively common, EAF is exceedingly rare.

The differential diagnosis of EAF includes Wegener granulomatosis (WG) [6, 29], granuloma faciale (GF) [6, 19, 20, 28], nasal NK/T-cell lymphoma, Churg-Strauss syndrome, and angiolymphoid hyperplasia with eosinophilia (ALHE) [1–4, 6, 10, 11, 14, 16, 20, 22, 25, 28–30]. EAF lacks manifestations of systemic disease. Its laboratory investigations are generally in normal ranges. The most important evidence comes from historical findings whether there was fibrinoid necrosis and granuloma formation or not. WG is a vasculitic process containing substantial necrobiosis and scattered foreign-body-type giant cells that can involve multiple anatomic sites, including the sinonasal cavity, orbit, and temporal bone. Positive C-ANCA may support a suspected diagnosis of WG [6, 29]. GF is an inflammatory cutaneous reaction. Patients with GF always present with purpuric plaques on the bridge of the nose and nasal deformity. Five earlier cases with concurrent EAF and GF have been reported. The histopathological appearances of EAF are very similar to GF, with onion-skin-like whorling of collagen fibrosis around small blood vessels. Some doctors even considered EAF as an extracutaneous lesion of GF. The natural histories of both diseases seem to be similar but our patient did not have any sign of skin lesion [3, 6, 15, 19, 20, 28]. Nasal NK/T-cell lymphoma exhibits soft-tissue thickening with or without necrosis of nasal cavity, facial subcutaneous, midline destruction, and extension into the nasopharynx [35]. Churg-Strauss syndrome is a kind of widespread necrotizing angiitis with granulomas. Eosinophilia and lung involvement are frequent. Asthma or other respiratory infection may precede the evidence of vasculitis [36, 37]. ALHE is an idiopathic condition that presents with papules, plaques, or nodules in the skin lesions in the periauricular region, forehead, or scalp. ALHE is marked by a proliferation of blood vessels with distinctive large endothelial cells. These blood vessels are accompanied by a characteristic inflammatory cell infiltration which includes a lot of eosinophils [38].

Until now, although consensuses did not reach the treatment strategy of EAF, surgery excision was performed in almost all reported case reports [1, 3, 13, 14, 18, 26, 27, 30]. EAF is generally accepted as a nonmalignant tumor-like lesion without risk of distant metastasis. However, it still has the ability to progressively destruct the local adjacent bone and the functions of an organ. It might block the nasolacrimal duct or impair ocular movement once the orbit was involved. Trauma or irritation to the nasal mucosa might contribute to the development of the tumor lesion. Incomplete surgical resection at the first time or even biopsy might play a negative role to stimulate and accelerate the tumor growth. Our patient received local excision in another ENT center for ventilation two years ago, but soon she almost could not breathe through the nose. We totally fished out tumor tissues and resected damaged cartilage until surrounding normal mucosa could be clearly identified. Complete removal of lesions and involved tissues might be the key to prevent EAF recurrence. The intranasal steroids may also perform a significant role despite that the potential mechanism is not clear. 

## 4. Conclusion

EAF is a rare benign disorder which may be progressive and cause local tissue destruction. Radiological imaging and historical findings contribute to the diagnosis of EAF. Laboratory results may be nonspecific. The allergic and eosinophilic infiltration may represent an abnormal inflammatory response to nonspecific stimulus in predisposed individuals. It is important to prevent tumor from recurrence by totally resecting the involved tissues.
